# Community buprenorphine continuation post-release following extended release vs. sublingual buprenorphine during incarceration: a pilot project in Maine

**DOI:** 10.1186/s40352-024-00281-w

**Published:** 2024-06-28

**Authors:** Alane B. O’Connor, Catherine Gelsinger, Sadie M. Donovan, Jessica Marshall, Katherine A. Ahrens

**Affiliations:** 1 Somerset County Jail, 131 E. Madison Rd, Madison, ME 04950 USA; 2 Muskie School of Public Service, University of Southern Maine, Portland, ME USA

**Keywords:** Extended-release buprenorphine, Incarceration, Rural jail, Opioid use disorder

## Abstract

**Background:**

The aim of our study was to evaluate the post-release outcomes of incarcerated individuals with opioid use disorder (OUD) treated with extended-release buprenorphine (XRB) in a rural county jail. Administrative data were collected from a pilot program within a jail in Maine that introduced XRB treatment in 2022 and a comparable jail utilizing sublingual buprenorphine (SLB) during the same period to compare post-release outcomes. Log-binomial regression models were used to estimate the risk ratio (RR) and 95% confidence interval (CI) for jail use of XRB vs. SLB on post-release community buprenorphine continuation.

**Results:**

From September 2022 to September 2023, 70 individuals who received XRB were released from the pilot jail and 130 individuals who received SLB were released from the comparison jail. After adjusting for age, sex, and buprenorphine use at entry to jail, individuals released from the pilot jail were almost 3 times (adjusted RR = 2.67, 95% CI 1.84, 3.88) as likely to continue community buprenorphine treatment post-release relative to the comparison jail. In addition, utilization of XRB allowed for expanded access to OUD treatment, was well tolerated, and reduced medication diversion.

**Conclusions:**

In this pilot program in Maine, XRB treatment during incarceration was associated with higher post-release community buprenorphine continuation when compared to individuals treated with SLB. These findings provide strong evidence for the superiority of XRB vs. SLB for the treatment of OUD in jail settings.

## Highlights

**•** Utilization of extended-release buprenorphine (XRB) in jail reduced diversion.
**•** Using XRB reduced staffing demands and expanded access to treatment.
**•** XRB may have reduced post-release overdose deaths.
**•** Those treated with XRB were 3 times as likely to continue buprenorphine post-release.

## Background

Substance use disorder (SUD) is common among the incarcerated population in the US (NCASA, 2010). According to the 2016 National Inmate Survey, 47% of adults in state and federal corrections facilities met the criteria for SUD (Maruschak et al., 2021). Further, individuals with opioid use disorder (OUD) are overrepresented in correctional facilities due to the connection between opioid misuse and use disorder and criminal justice involvement (Winkelman et al., 2018). However, few individuals with OUD are able to access treatment in jails and, therefore, experience a decrease in opioid tolerance while incarcerated, placing them at high risk for fatal overdose after release, especially within the first 2 weeks (Binswanger et al., 2007; Merrall et al., 2010; Ranapurwala et al., 2018).

Medication for OUD (MOUD) remains the most effective OUD treatment and includes buprenorphine and methadone (both agonists) and extended-release naltrexone (an antagonist) (Crotty et al., 2020; NASEM, 2019). MOUD treatment reduces opioid use and cravings, improves health outcomes, and saves lives (NASEM, 2019; Sordo et al., 2017). The American Society of Addiction Medicine practice guidelines recommend the provision of MOUD in all correctional settings (Crotty et al., 2020) and this has been shown to increase treatment engagement in the community, decrease recidivism, and reduce post-release drug use and overdose deaths (Cates & Brown, 2023; Evans et al., 2022; Green et al., 2018; Lim et al., 2023).

Despite the extensive evidence supporting the use of MOUD in correctional settings, only a small percentage of facilities in the US offer MOUD treatment and, when offered, access is often limited to select populations (e.g., those pregnant and/or already on MOUD at jail entry) (Weizman et al., 2021). Sublingual buprenorphine (SLB) is the standard treatment in most US jails that offer MOUD; it is taken daily and has a half-life of approximately 24 to 42 h (Indivior, 2023). Very few jails offer extended-release buprenorphine (XRB), which is given monthly by subcutaneous injection (Heidbreder et al., 2023; Homans et al., 2023). While XRB is effective and well-tolerated, facilities often cite the cost of the medication as a primary barrier to implementation in a correctional setting (Berk et al., 2022; Lee et al., 2021; Lintzeris et al., 2021; Wong et al., 2022). Still, XRB overcomes some of the other key barriers to MOUD implementation in jails and prisons such as diversion concerns, increased need for medical and security staff resources, and stigma toward those with OUD (Cheng et al., 2022; Lee et al., 2021; Martin et al., 2022). Additionally, XRB has the potential to decrease the risk of post-release drug overdose by acting as a longer “bridge” to community buprenorphine treatment programs because the medication is therapeutic for approximately 26 to 44 days (Berk et al., 2022; Indivior, 2022).

Maine, a rural state, has been deeply impacted by the opioid crisis and has experienced higher opioid-related overdose death rates when compared with the national average (e.g. 70% higher in 2021) (Kaiser Family Foundation, 2023b). Until 2022, many Maine jails had only been able to offer buprenorphine (using SLB) to those already in community buprenorphine treatment at the time of their incarceration. However, in early 2022 one rural jail sought to expand access to treatment to all incarcerated individuals with OUD. Using US Health Research and Service Administration (HRSA) Rural Communities Opioid Response Program grant funding, obtained by the County’s public health department, the Sheriff hired a director of addiction medicine (study author AO) who had nearly two decades of experience treating individuals with SUD. XRB had not previously been used in the jail but was the only option that allowed for expanding buprenorphine treatment to all individuals with OUD given the staffing limitations at the jail. While designing an XRB pilot project, the jail staff worked closely with Maine’s Director of Opioid Response as well as Maine’s Office of Behavioral Health to secure funding from Maine’s Opioid Use Disorder Prevention and Treatment Fund to support the cost of purchasing XRB. County commissioners also designated their counties’ opioid settlement funds toward the project. Administrators at the pilot jail sought to compare the post-release outcomes of individuals treated with XRB with those from a comparable rural county jail in Maine that used only SLB during the same timeframe.

## Methods

### Pilot jail and comparison jail

The study population was comprised of two regional jails located in rural settings in Maine. The average daily population of Maine jails is 1700 (17% female) and the average time in jail is 19.6 days (Zeng & Minton, 2021). The average monthly inmate census at the comparison jail was approximately 15% higher than the pilot jail. Medical and behavioral health care in both jails was provided by the same medical vendor and pre-release planning and referrals were similar in both facilities. Both jails had case managers and social work staff who assisted patients in reactivating insurance and accessing community SUD treatment options post-release. Post-release OUD treatment availability in both communities was typical for rural Maine (e.g., small local in-person buprenorphine treatment programs, state-wide telehealth buprenorphine options, nearest methadone clinic more than 20 miles away). At both jails, those with verifiable adherence with methadone at jail entry were continued on methadone but this was a small number of individuals (only 3 at the pilot jail). Those without verifiable adherence were offered buprenorphine treatment. In both jails, only a small percentage of those incarcerated (about 10%) were serving a sentence with a scheduled release date. The remainder were pre-trial or being held on a bail revocation or probation hold and had uncertain release dates.

### Extended-release buprenorphine pilot project

The pilot jail began exclusively offering XRB treatment for individuals with OUD in September 2022 and treatment coverage was expanded over the course of several months. Initially those already in treatment with SLB (i.e., had a community buprenorphine provider upon jail entry) were transitioned to XRB. Next, individuals with OUD who were serving a sentence with a scheduled release date were offered XRB. Finally, all individuals with OUD (both those already in custody as well as individuals entering the facility) were offered treatment with XRB after arraignment (typically 48–72 h after entry). Treatment was offered to all individuals in a specific housing unit at the same time and a new housing unit was brought online each week. Four individuals diagnosed with OUD in the pilot jail declined XRB treatment.

XRB, which remains therapeutic for approximately 26 to 44 days (median 35 days), was administered monthly by subcutaneous injection. The selection process and rapid access induction protocol for administration of XRB is described in detail in Table 1. This protocol was created by the jail’s addiction medicine director to ensure timely access to treatment in the setting of short jail stays and unpredictable release dates. Individuals were screened for OUD during the medical intake (which occurred within 24 h of entry) and again after arraignment (if they previously screened negative). Those screening positive were assessed by jail medical or counseling staff and then referred for treatment if medically indicated. While awaiting buprenorphine treatment, opioid withdrawal was treated with medications (e.g., non-opioid medications such as clonidine and muscle relaxants) and other non-pharmacologic options (e.g., more blankets, special diet) as needed. SLB was typically initiated a few days prior to the first scheduled XRB injection. Initial XRB injections were given by the jail’s medical staff; subsequent XRB injections were administered by nursing staff. Most individuals not in buprenorphine treatment in the community upon arrival received their initial XRB injection about seven days after jail entry.

**Table 1:** Protocol for administration of extended-release buprenorphine at pilot jail

**Patient selection to determine if patient has opioid use disorder and last opioid exposure**			
*Selection tool*	*If yes*,	*If no*,	
Screening for opioid use disorder	If screening indicates opioid use disorder, then proceed with XRB induction protocol	If screening indicates no opioid use disorder, then do not proceed with XRB induction protocol	
Prescription Monitoring Program (PMP) verification of most recent opioid prescription	If PMP indicates buprenorphine prescriptions within past 30 days, then use most recent day of buprenorphine treatment to determine the day of last opioid exposure	If PMP indicates most recent buprenorphine prescription not within past 30 days, then assume most recent opioid exposure more than 7 days ago, unless the urine toxicology test is positive for an opioid (e.g., fentanyl, buprenorphine, methadone, etc.)	
Urine toxicology test	If urine toxicology test is positive for an opioid, assume the last opioid exposure less than or equal to 7 days ago	If urine toxicology test is negative for opioids, assume last opioid exposure was more than 7 days ago	
Pregnancy test (females only)	If pregnancy test is positive, do not initiate XRB induction protocol	If pregnancy test is negative, proceed with XRB induction protocol	
**Extended-release buprenorphine induction protocol**:			
*Last opioid exposure*	*Day 1*	*Day 2*	*Day 3*
More than 30 days ago+	2 mg sublingual buprenorphine	4 mg sublingual buprenorphine	XRB 100 mg
More than 7 but less than or equal to 30 days ago	4 mg sublingual buprenorphine	8 mg sublingual buprenorphine	XRB 300 mg
Less than or equal to 7 days ago	8 mg sublingual buprenorphine	XRB 300 mg	
Ongoing XRB treatment			
If individuals received XRB 300 mg as the first dose, they received another 300 mg dose the next month and then 100 mg monthly thereafter. Those initiated on 100 mg received 100 mg monthly for the duration of their incarceration. +This situation only applied to those already in custody when the program was implemented and those transferring to the pilot jail from a correctional facility that did not offer buprenorphine.			

XRB = Extended-release buprenorphine

The comparison jail exclusively used SLB, which was the standard of care for incarcerated individuals with OUD throughout the state of Maine at the time of the pilot project. The comparison jail sample was limited to individuals incarcerated for at least 5 days because this was the minimum days that someone in the pilot jail needed to be incarcerated to proceed through the rapid induction protocol for XRB. At the comparison jail, medical staff electronically faxed a 7-day SLB prescription to the patient’s preferred pharmacy on the day of release. For up to 28 days, jail medical staff renewed this prescription in 7-day intervals if the patient contacted the jail’s medical facility. No individuals who declined SLB at the comparison jail were identified through the chart review.

### Characteristics of incarcerated individuals

Clinical staff manually abstracted demographics, SUD information, and buprenorphine treatment history from paper and electronic jail records. Age (at entry), sex, and buprenorphine treatment status upon jail entry (from another facility or the community) were abstracted from both jails; race/ethnicity, educational attainment, employment status, and OUD severity (based on Drug Abuse Screening Test [DAST-10] scores) were abstracted from the pilot jail only. At the pilot jail, clinical staff abstracted the number of XRB doses administered per person in the jail and the days between the last administration of XRB and release from jail. At the comparison jail, clinical staff abstracted the number of days treated with SLB per person in the jail. For those individuals who were incarcerated more than once during the study period, only data from their first incarceration were included.

### Study outcome 1: community buprenorphine treatment continuation post-release

Clinical staff compiled information on buprenorphine prescriptions post-release. They reviewed the Maine Prescription Monitoring Program (PMP) information to identify whether a prescription was filled for SLB within 35 days of release from jail. This number of days was selected due to the maximum expected duration of therapeutic days of XRB (35 days if XRB was given on day of release). Clinical staff also reviewed medical claims (procedure codes Q9991 and Q9992) and pharmacy claims (NDC code 12,496,010,001) paid for by Maine’s Medicaid program to identify XRB administrations within 35 days of jail release. Methadone referral post-release was uncommon among incarcerated individuals at both jails, so methadone medical claims were not requested from Maine’s Medicaid program for review; in addition, methadone administration is not captured in the Maine PMP.

We created a dichotomous measure for post-release buprenorphine treatment continuation. For the pilot jail, this was obtaining either SLB or XRB while buprenorphine was still therapeutic from the last dose of XRB while incarcerated. To determine this threshold, we added 35 days to the last day of XRB administration that occurred within the jail. For the comparison jail, buprenorphine treatment continuation was defined as filling a SLB prescription or obtaining XRB from a community prescriber ≤ 2 days after release from jail *or* filling a SLB prescription from a jail provider ≤ 2 days after release and then filling a SLB prescription (or obtaining XRB) from a community prescriber ≤ 2 days after the final jail prescription expired.

### Study outcome 2: diversion, safety concerns, and XRB side effects

Clinical staff at the pilot jail reviewed medical and jail records to identify medication diversion, safety concerns, and side effects.

### Study outcome 3: mortality events post-release

In September 2023, clinical staff sent the names and dates of birth of individuals treated with XRB or SLB in their respective jails to the Office of Maine Data, Research, and Vital Statistics (DRVS). DRVS matched this information to death records of persons who resided in Maine and died in Maine during September 2022 to September 2023. If a person matched this list, the underlying cause of death and date of death were returned to clinical staff.

### Statistical analysis

We tabulated the demographic characteristics of individuals released from the pilot and comparison jails, separately, and calculated the mean number of XRB administrations per person at the pilot jail and days treated with SLB per person at the comparison jail. We used log-binomial regression models to estimate the risk ratio for XRB vs. SLB on community buprenorphine treatment continuation post-release, adjusting for individuals’ age (using 3-level age grouping), sex, and buprenorphine treatment (yes/no) upon entry to the jail.

### Ethical approval

The study was determined by the Institutional Review Board of the University of Southern Maine to not be human subject research. This determination was based on the study being a quality improvement project, intended to evaluate a new state pilot program rather than generate generalizable knowledge. Study authors at the University of Southern Maine performed the analysis using de-identified aggregated data. Denominator cell counts between 1 and 4 for non-missing category levels were suppressed from tables for privacy purposes. To further protect privacy, Maine’s leading medical ethicist reviewed and approved the pilot project prior to implementation.

## Results

There were 70 individuals who received XRB released from the pilot jail and 130 individuals who received SLB released from the comparison jail during September 2022 to September 2023 (Table 2). Individuals from the pilot jail had a mean age of 36 years, were 66% male, and were 97% non-Hispanic white. Individuals from the comparison jail had a mean age of 37 years and were 94% male (race/ethnicity not collected from comparison site). The smaller number of females at the comparison jail was because incarcerated females were often housed at other facilities due to security staffing shortages, a common practice among rural Maine jails.

**Table 2:** Characteristics of individuals with opioid use disorder incarcerated at the pilot jail and comparison jail between September 1, 2022-September 1, 2023

Characteristic	Pilot jail (XRB treatment)	*N*	Comparison jail (SLB treatment)	*N*
Total		70		130
Buprenorphine treatment in Jail				
Number of XRB administrations in jail per person, mean (SD)	2.1 (1.7)		─	
Days treated with SLB in jail per person, mean (SD)	─		75 (60.6)	
Age, mean (SD)	36 (8.1)		37 (8.2)	
Sex (%)				
Male	65.7	46	93.9	122
Female	34.3	24	6.2	8
Race/Ethnicity (%)				
White	97.1	68	─	
Black/Hispanic/Native American	Suppressed	Suppressed	─	
Education Level (%)				
Missing	17.1	12		
9th to 12th grade	10.0	7	─	
GED	24.3	17	─	
High school graduate	24.3	17	─	
Post-secondary	24.3	17	─	
Employed upon jail entry (%)				
Missing	32.9	12	─	
Yes	30.0	21	─	
No	52.9	37	─	
DAST Score (%)^a^				
Missing	32.9	23		
Low/moderate	7.1	5	─	
Substantial/severe	60.0	42	─	
Buprenorphine on jail entry (%)^b^				
Yes	30.0	21	36.9	48
No	70.0	49	63.1	82

─ Data not collected for group

*Abbreviations* DAST = Drug Abuse Screening Test; GED = General Educational Development test; MOUD = Medication for Opioid Use Disorder; SD = Standard Deviation; SLB = sublingual buprenorphine; XRB = extended-release buprenorphine

^a^DAST Score: Low = 1–2; Moderate = 3–5; Substantial = 6–8; Severe = 9–10

^B^Buprenorphine on Entry = Active buprenorphine treatment at jail entry (from another corrections facility or community)

At the pilot jail, 30% of individuals were receiving buprenorphine treatment at the time of entry; at the comparison jail, 37% were receiving buprenorphine treatment. At the pilot jail (among those with non-missing data), 88% of individuals had graduated from high school or had passed the General Educational Development test, 63% were unemployed at jail entry, and 89% met the criteria for a substantial or severe SUD. The mean number of XRB administrations per person at the pilot jail was 2.1 (equivalent to 63 days of treatment assuming 30 days per administration), and the mean days of those treated with SLB per person at the comparison jail was 75.

### Study outcome 1: community buprenorphine treatment continuation post-release

Approximately 67% (47 of 70) of individuals released from the pilot jail continued buprenorphine treatment post-release without interruption; in the comparison jail, this was only 23% (30 of 130) (Table 3). A higher percentage of those in vs. not in buprenorphine treatment at time of jail entry continued buprenorphine treatment post-release at both the pilot (91% vs. 57%) and comparison jail (33% vs. 17%); patterns by age group were distinct at the pilot (youngest age group was more likely to continue buprenorphine treatment post-release) and comparison jails (oldest group was more likely) and did not differ by sex.

**Table 3:** Post-release community buprenorphine continuation at pilot jail and comparison jail by individuals’ characteristics between September 1, 2022-September 1, 2023

Characteristic	Pilot jail (XRB treatment)	*N*	Comparison jail (SLB treatment)	*N*
Post-release Community Buprenorphine Continuation (%)	67.1	47/70	23.1	30/130
Age group (%)				
18–35	75.8	25/33	19.1	12/63
36–45	59.3	16/27	24.0	12/50
46–65	60.0	6/10	35.3	6/17
Sex (%)				
Male	67.4	31/46	23.0	28/122
Female	66.7	16/24	25.0	2/8
Race/Ethnicity (%)				
White	66.2	45/68	─	
Black/Hispanic/Native American	Suppressed	Suppressed	─	
Education level (%)				
Missing	66.7	8/12		
9th to 12th grade	57.1	4/7	─	
GED	70.6	12/17		
High school graduate	70.6	12/17	─	
Post-secondary	64.7	11/17	─	
Employed upon jail entry (%)				
Missing	66.7	8/12		
Yes	61.9	13/21	─	
No	70.3	26/37	─	
DAST score (%)^a^				
Missing	56.5	13/23	─	
Low/moderate	60.0	3/5	─	
Substantial/severe	73.0	31/42	─	
Buprenorphine on entry (%)^b^				
Yes	90.5	19/21	33.3	16/48
No	57.1	28/49	17.1	14/82

─ Data not collected for group

*Abbreviations* DAST = Drug Abuse Screening Test; GED = General Educational Development test; MOUD = Medication for Opioid Use Disorder; SD = Standard Deviation; SLB = sublingual buprenorphine; XRB = extended-release buprenorphine

^a^ DAST Score: Low = 1–2; Moderate = 3–5; Substantial = 6–8; Severe = 9–10

^b^ Buprenorphine on Entry = Active buprenorphine treatment at jail entry (from another corrections facility or community)

**Fig. 1:**
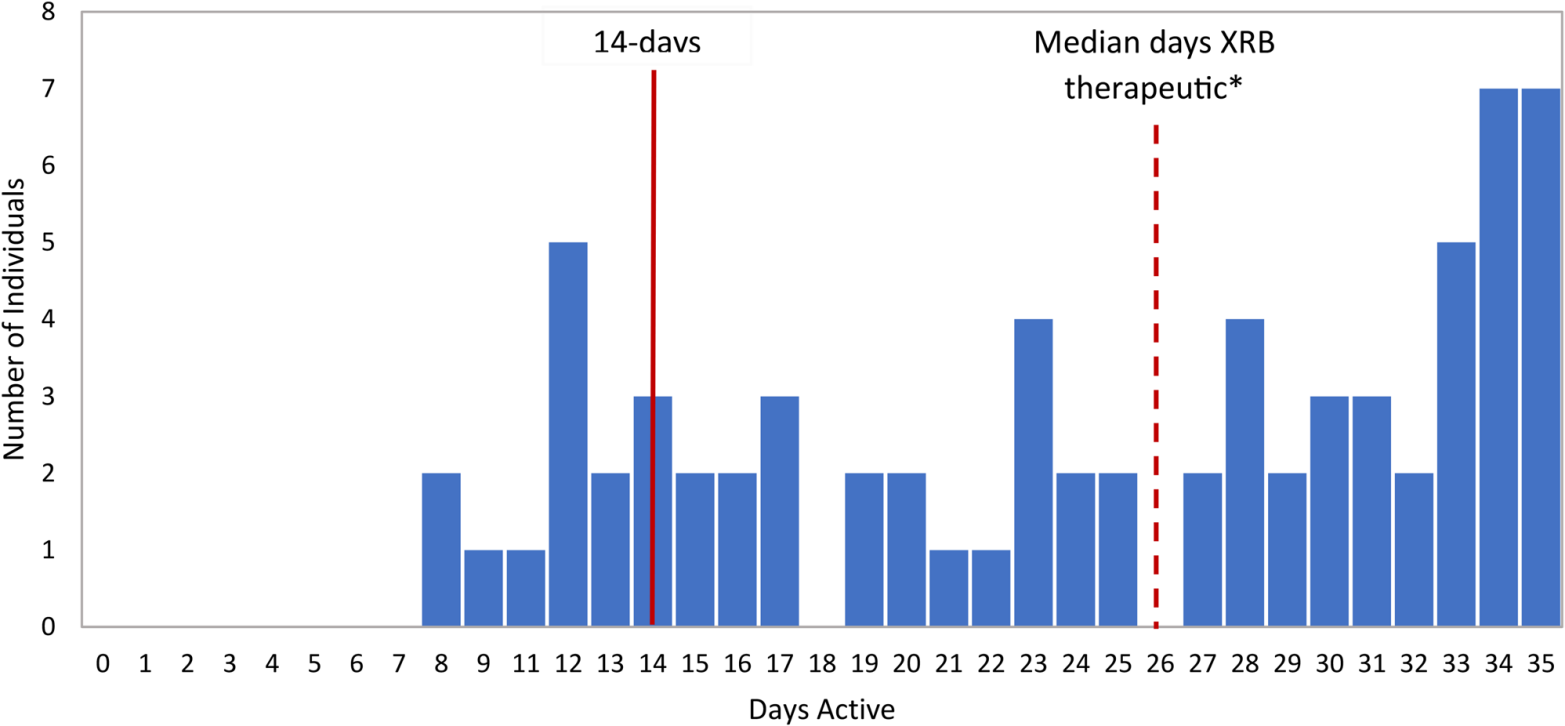
Distribution of days post-release that extended-release buprenorphine was therapeutic for pilot jail individuals (*n* = 70). *Median days therapeutic was 26, which was the arithmetic average of the middle two days (day 25 and day 27)

**Fig. 2:**
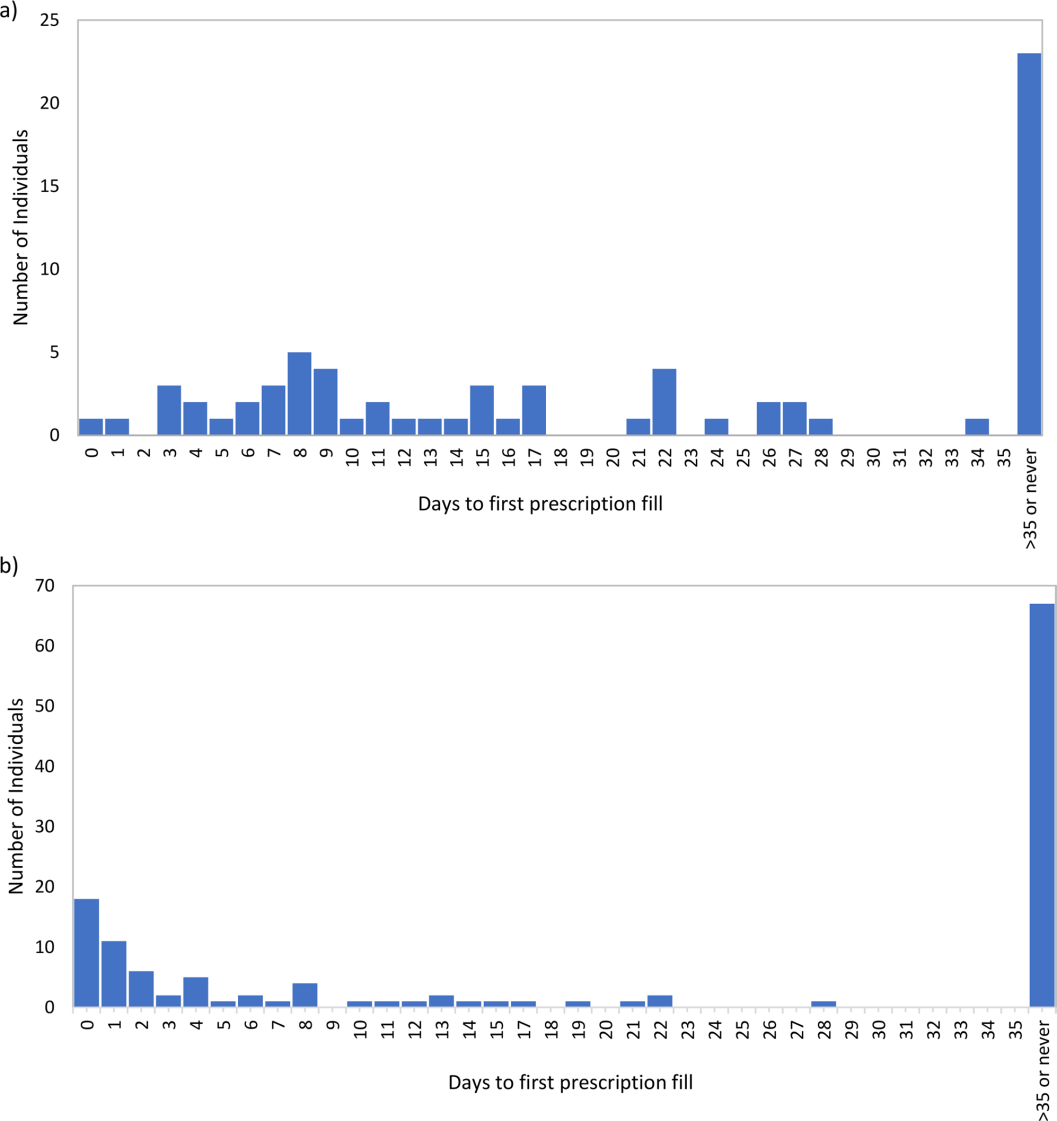
Distribution of days post-release to first prescription fill for buprenorphine. (**a**) Pilot jail, *n* = 70. Within the first 35 days: mean = 13.1, minimum = 0, maximum = 34. (**b**) Comparison jail, *n* = 130. Within the first 35 days: mean = 4.4, minimum = 0, maximum = 28

The median number of days that buprenorphine was therapeutic in individuals released from the pilot jail was 26 (interquartile range from 16 to 33), and 59 of 70 (84%) individuals had therapeutic levels for at least 14 days post-release (Fig. 1). Individuals released from the pilot jail had a more uniform distribution of days to first prescription filled (or XRB injection) within the first 35 days (Fig. 2a) as compared to individuals released from the comparison jail, which was right skewed (Fig. 2b). In addition, the mean number of days (within the first 35 days) until the first prescription was filled (or XRB injection) for individuals released from the pilot jail was 13.1 days, which was higher than the corresponding mean number of days for individuals released from the comparison jail (4.4 days).

When compared to the jail offering SLB, individuals treated with XRB at the pilot jail were almost 3 times (RR = 2.91, 95% CI 2.04, 4.15) as likely to continue buprenorphine in the community post-release—that is, receive buprenorphine treatment from a community provider while buprenorphine was still therapeutic (Table 4). After adjusting for 3-level age group, sex, and buprenorphine at entry to jail (yes/no), the risk ratio remained similar (adjusted RR = 2.67, 95% CI 1.84, 3.88).

**Table 4:** Association between buprenorphine treatment type at release and post-release community buprenorphine continuation at pilot jail and comparison jail between September 1, 2022-September 1, 2023

Treatment type at release	*N*	(%)	Risk ratio	95% CI	*p*-value	Adjusted risk ratio^a^	95% CI	*p*-value
Pilot jail (XRB treatment)	70	67.1	2.91	2.04, 4.15	< 0.0001	2.67	1.84, 3.88	< 0.0001
Comparison jail (SLB treatment)	130	23.1	*Reference*			*Reference*		

*Abbreviations* CI = confidence interval; SLB = sublingual buprenorphine; XRB = extended-release buprenorphine

^a^ Adjusted for 3-level age group, sex, and buprenorphine treatment on entry to jail

### Study outcome 2: diversion, safety concerns, and XRB side effects

None of the patients treated with XRB experienced an allergic reaction, had complications at the injection site, or required treatment in a hospital related to their SUD. The most common side effects were constipation and peripheral edema, consistent with other forms of buprenorphine (Indivior UK Limited, 2023). No patients discontinued XRB in the jail due to side effects. There were no documented cases of acute precipitated withdrawal during the initiation process. No diversion events of XRB that had been administered into the abdomen were reported or suspected. Shortly after the pilot began, medical staff realized that band aids covering the XRB injection site were being traded and sold in the housing units. Staff began removing the band aid while the individual was still under medical supervision and there were no further reports of diversion.

### Study outcome 3: mortality post-release

There were 4 deaths post-release from the comparison jail and zero deaths from the pilot jail as of September 2023. The four decedents were men and ranged in age from 35 to 50 years. Three deaths were due to unintentional drug overdose and one death was due to suicide. The mean day of death post-release for those that died of drug overdose was 71. None of the decedents received buprenorphine treatment (with either XRB or SLB) from a community prescriber post-release.

## Discussion

This evaluation of a pilot project using XRB for the treatment of OUD in a rural Maine jail found two-thirds of individuals continued buprenorphine treatment post-release. Additionally, about eight in ten of those individuals had therapeutic levels of buprenorphine for at least 14 days post-release; this two-week window is when the risk of overdose post-release is the highest (Binswanger et al., 2013; Merrall et al., 2010). For individuals at the comparison jail, only a quarter continued buprenorphine treatment post-release. This nearly 3-fold difference suggests that individuals who receive XRB prior to release, as compared to SLB, which is the standard of care in Maine, have a lower risk of return to illicit opioid use and overdose post-release. We found zero deaths post-release among the 70 participants treated with XRB, as compared with 4 deaths post-release among the 130 participants treated with SLB.

At the pilot jail, XRB was well tolerated and addressed many of the common barriers to MOUD implementation in correctional facilities, particularly diversion and staffing concerns. Monthly (rather than daily) visits to the jail’s medical facility required fewer hours of security and medical staff’s time and enabled the pilot jail to treat all individuals with OUD, not only those in buprenorphine treatment prior to incarceration. Anecdotally, jail administrators also noted less stress among both staff and inmates with fewer daily trips to the medical facility and when diversion was not a concern within the housing units.

Better treatment retention for the pilot jail site could be because XRB treatment, as opposed to SLB, is easier to manage during the post-release time of competing financial, legal, health, and social stressors (Martin, 2018). In addition, SLB prescriptions require active health insurance, which is often discontinued if the individual misses his/her 6-month review while incarcerated and has an unexpectedly early release that precludes appropriate planning. In addition, the higher number of inmates and higher percentage of male inmates at the comparison jail could explain the higher number of deaths observed post-release.

Our post-release buprenorphine continuation findings are consistent with previous studies on the use of XRB within correctional facilities. Martin et al. found that 70% (23 of 33) of incarcerated individuals who were treated with XRB continued with buprenorphine treatment in the community post-release. However, average time to first medication fill was 23.7 days in the Martin study, which was longer than we found for the pilot jail (mean = 13.1 days, among those with fills by day 35) (Martin et al., 2022). A small, randomized trial with 52 participants from New York City found community buprenorphine treatment retention at 8-weeks post-release was twice as high (69% vs. 35%) for participants treated with XRB compared with SLB prior to release (Lee et al., 2021). The higher treatment retention in the New York City study comparison group (35%) relative to our comparison jail (23%) may be due to their randomized controlled trial study design (with incentives for participating) versus our observational study design or our rural jail setting.

Our study’s finding of lower mortality for the XRB treatment group, which provided longer therapeutic levels of buprenorphine post-release than for those on SLB, are broadly consistent with studies examining OUD treatment in jails and subsequent mortality. For example, an observational study in a New York City jail found that individuals who received SLB or methadone prior to release had an 80% reduction in post-release overdose death compared with those who did not receive MOUD prior to release (Lim et al., 2023). To our knowledge, no study has yet found that XRB vs. SLB may result in fewer overdose deaths, as we have found.

### Policy implications

Combined with findings from other studies of MOUD treatment at the time of jail release, our study findings highlight the need for access to OUD treatment for all incarcerated individuals with OUD. The correctional setting can provide an important opportunity to engage individuals in treatment due to the strong associations between SUD and criminal justice involvement (Hoover et al., 2023; Winkelman et al., 2018). In addition to providing MOUD during incarceration—including XRB, other policy changes are needed to increase access to care and improve care transitions upon release to the community. A recent analysis of survey results from the Rural Opioid Initiative found that individuals who had been recently incarcerated were more likely to report having difficulty accessing MOUD than those without a history of recent incarceration (Hoover et al., 2023). The study also found that recent incarceration was associated with lower rates of health insurance coverage, which could contribute to gaps in post-release treatment and medication adherence during a transition period in which individuals may experience high levels of stress and uncertainty (Hoover et al., 2023).

Currently, federal law excludes individuals from Medicaid coverage while incarcerated, potentially exacerbating these gaps in treatment. According to a report to Congress from the Office of the Assistant Secretary for Planning and Evaluation, allowing pre-release Medicaid coverage for individuals via 1115 Demonstration waivers is a “significant potential opportunity to promote access to and continuity of health coverage and care for returning community members” (p. 35) (Feinberg et al., 2023). As of December 21st 2023, 19 states have applied for Sect. 1115 Medicaid waivers for justice-involved individuals and three waivers have been approved (Kaiser Family Foundation, 2023a). Even if states are granted 1115 Demonstration waivers allowing pre-release Medicaid coverage, the high upfront cost of XRB during incarceration may continue to be a barrier. One solution could involve eliminating the Medicaid exclusion for incarcerated individuals altogether, a change that would also encourage consistency in SUD treatment within correctional facilities nationally (Hoover et al., 2023).

While the Maine Opioid Response Clinical Advisory Committee recommended expanding community access to XRB (O’Connor et al., 2022) and Medicaid in Maine covers XRB for individuals recently incarcerated (Maine Department of Health and Human Serivces, 2023), access to ongoing community XRB treatment can be challenging, especially in rural and medically underserved areas. A review of post-release access to XRB during the study period found that 12 of the 70 patients enrolled in the pilot (17%) continued XRB post-release. However, several patients treated in the XRB pilot returned to the facility post-release seeking assistance finding an XRB prescriber after reporting that their community prescriber was offering only SLB. Half (3 of 6) of the individuals treated with XRB during incarceration and then released into a small community recovery court (where both XRB and SLB are available) remained on XRB post-release. At the comparison jail, only 4 of the 130 patients (3%) utilized XRB post-release during the study period.

### Strengths and limitations

To the best of our knowledge, ours is the first observational study of XRB used in a correctional facility in a non-urban setting. Our sample is also considerably larger than previous studies of XRB in correctional facilities, and we compared our post-release outcomes to those of a comparison jail. Limitations of our study include the lack of access to post-release commercial claims for XRB and methadone treatment data, which would have allowed us to comprehensively identify the first community MOUD treatment engagement post-release. While we believe that pre-release planning and OUD treatment referrals were similar at both facilities, there may be differences that we were not able to capture that influenced our results.

Our study evaluation did not capture data related to cost or recidivism outcomes; however, future studies of this XRB pilot program aim to collect these data. Our study’s mortality data are limited because they only included deaths that occurred in Maine for individuals who resided in Maine at the time of death, and follow-up times ranged from one month (for persons released from jail in September 2023) to 12 months (for persons released in September 2022). Our study only measured community buprenorphine treatment continuation post-release, defined as days while buprenorphine was still therapeutic from the last dose (which was on average longer for XRB than for SLB). Future studies could compare community buprenorphine treatment at fixed time points further out, like 30 days, 60 days, 90 days, 6 months, etc., to obtain a more comprehensive picture of longer-term community buprenorphine treatment by XRB or SLB treatment in jail. Finally, our study was observational, so we cannot make causal claims about XRB vs. SLB treatment on post-release outcomes.

## Conclusions

In conclusion, utilizing XRB in a correctional facility was associated with a higher rate of buprenorphine treatment continuation post-release when compared to individuals released from a jail offering SLB. XRB’s longer duration of therapeutic buprenorphine likely reduces the risk of return to use immediately post-release when the overdose risk is highest. XRB was well-tolerated, reduced staffing burden associated with daily SLB administration and dramatically reduced diversion suggesting that it may be a superior option for treating individuals with OUD in correctional facilities.

## Data Availability

The data that support the findings of this study are not openly available due to reasons of sensitivity but de-identified data is available from the corresponding author upon reasonable request with the approval of the Sheriff’s office.
